# Prospective surveillance for intussusception in Indian children aged under two years at nineteen tertiary care hospitals

**DOI:** 10.1186/s12887-020-02293-5

**Published:** 2020-09-01

**Authors:** The INCLEN Intussusception Surveillance Network Study Group, Manoja Kumar Das, Narendra Kumar Arora, Bini Gupta, Apoorva Sharan, Mahesh K. Aggarwal, Pradeep Haldar, Patrick L. F. Zuber, Jan Bonhoeffer, Arindam Ray, Ashish Wakhlu, Bhadresh R. Vyas, Javeed Iqbal Bhat, Jayanta K. Goswami, John Mathai, K. Kameswari, Lalit Bharadia, Lalit Sankhe, M. K. Ajayakumar, Neelam Mohan, Pradeep K. Jena, Rachita Sarangi, Rashmi Shad, Sanjib K. Debbarma, J. Shyamala, Simmi K. Ratan, Suman Sarkar, Vijayendra Kumar, Yoga Nagender, Anand P. Dubey, Atul Gupta, Bashir Ahmad Charoo, Bikasha Bihary Tripathy, Cenita J. Sam, G. Rajendra Prasad, Gowhar Nazir Mufti, Harish Kumar. S., Harsh Trivedi, Jimmy Shad, Jothilakshmi. K., Sharmila. K., Kaushik Lahiri, Meera Luthra, Nihar Ranjan Sarkar, Padmalatha. P., Pavai Arunachalam, Rakesh Kumar, Ruchirendu Sarkar, S. S. G. Mohapatra, Santhosh Kumar. A., Saurabh Garge, Subrat Kumar Sahoo, Sunil K. Ghosh, Sushant Mane, Christine G. Maure

**Affiliations:** The INCLEN Trust International, F1/5, Okhla Industrial Area, Phase 1, New Delhi, 110020 India

**Keywords:** Intussusception, Prospective surveillance, Children, Epidemiology, Weaning food, Sociodemography, Rotavirus vaccine, India

## Abstract

**Background:**

India introduced rotavirus vaccines (RVV, monovalent, Rotavac™ and pentavalent, Rotasiil™) in April 2016 with 6, 10 and 14 weeks schedule and expanded countrywide in phases. We describe the epidemiology of intussusception among children aged 2–23 months in India.

**Methods:**

The prospective surveillance at 19 nationally representative sentinel hospitals from four regions recruited children with intussusception from April 2016 to September 2017. Data on sociodemography, immunization, clinical, treatment and outcome were collected. Along with descriptive analysis, key parameters between four regions were compared using Chi-Square/Fisher’s exact/Mann–Whitney U/Kruskal-Wallis tests. The pre- and post-RVV periods were compared to estimate the risk ratios.

**Results:**

Six hundred twenty-one children with intussusception from South (*n* = 262), East (*n* = 190), North (*n* = 136) and West (*n* = 33) regions were recruited. Majority (*n* = 465, 74.8%) were infants (40.0% aged 4–7 months) with median age 8 months (IQR 5, 13 months), predominantly males (*n* = 408, 65.7%) and half (*n* = 311, 50.0%) occurred during March–June months. A shorter interval between weaning and intussusception was observed for ragi based food (median 1 month, IQR 0–4.2 months) compared to rice (median 4 months, IQR 1–9 months) and wheat (median 3 months, IQR 1–7 months) based food (*p* < 0.01). Abdominal pain or excessive crying (82.8%), vomiting (72.6%), and bloody stool (58.1%) were the leading symptoms. Classical triad (abdominal pain, vomiting and bloody stool) was observed in 34.8% cases (24.4 to 45.8% across regions). 95.3% of the cases were diagnosed by ultrasound. 49.3% (10.5 to 82.4% across regions) cases were managed by reduction, 39.5% (11.5 to 71.1% across regions) cases underwent surgery and 11.1% spontaneously resolved. Eleven (1.8%) cases died. 89.1% cases met Brighton criteria level 1 and 7.6% met Level 2. RVV was received by 12 cases within 1–21 days prior to intussusception. No increase in case load (RR = 0.44; 95% CI 0.22–1.18) or case ratio (RR = 0.5; 95% CI 0.3–1.2) was observed after RVV introduction in select sites.

**Conclusions:**

Intussusception cases were observed across all sites, although there were variations in cases, presentation and mode of management. The high case load age coincided with age of the RVV third dose. The association with ragi based weaning food in intussusception needs further evaluation.

## Background

To reduce the rotavirus diarrhoea related childhood deaths, the World Health Organization (WHO) has recommended introduction of rotavirus vaccine (RVV) in national immunization programmes (NIPs). Some increased risk of intussusception after the first (relative risk, RR: 4.7–13.8) and second (RR: 1.3–5.3) doses of RVVs was reported from several countries (Mexico, Brazil, Australia, United Kingdom, United States, Spain and Singapore) [1–8]. But no increased risk of intussusception after any dose of RVV was observed in some countries (Ethiopia, Ghana, Kenya, Malawi, Tanzania, Zambia, Zimbabwe and South Africa) [9, 10]. The impact of RVV on diarrhoea morbidity and mortality outweigh the risk of intussusception and associated mortality [11]. In view of the concern about intussusception, documentation of baseline and monitoring following RVV introduction have been recommended [12]. India introduced RVV into the NIP in April 2016 and by 2019 expanded countrywide in four phases [13]. Two types of RVV are used in NIP, Rotavac™ (RV1-116E; Bharat Biotech) in 26 states/union territories and Rotasill™ (RV5; Serum. Institute of India) in 11 states/union territories, both follow 6, 10, 14 weeks schedule. Intussusception is an acute severe clinical condition occurring mostly during infancy, which overlaps with the age of primary vaccinations. The nationally representative background epidemiology of intussusception in India is not clear. The reports from India primarily included retrospective data and considerably varied in the epidemiology, presentation and management [14, 15]. Information on population level rate in India is limited and the reported incidences of intussusception requiring hospitalization varied from 17.7 (95% CI 5.9–41.4) in Delhi (North India) to 254 (95% CI 102, 524) cases per 100,000 child-years in Vellore (South India) [16, 17]. The incidence of intussusception vary widely globally, across the different high and low-middle income countries [18]. The reasons for the variations are unknown. There is no information from India regarding the regional variation in intussusception epidemiology. Thus, documentation of the intussusception epidemiology prior to RVV introduction to establish a reliable baseline for monitoring the trend over time and identify potential risk factors was needed to support the vaccine safety surveillance efforts [19, 20]. Under the vaccine safety surveillance effort, we describe the epidemiology, clinical characteristics of intussusception among children aged under-2 years seeking hospital care in India and initial changes with RVV introduction documented through a nationally representative sentinel surveillance network.

## Methods

### Study area and participating hospitals

This prospective active hospital-based sentinel surveillance was conducted over 18 months, at 19 nationally representative tertiary care hospitals (Supplementary Figure 1). From the four regions, 3–6 hospitals per region including medical colleges and private-sector hospitals (North region- 5 sites, 3 public and 2 private; South region- 5 sites, 2 public and 3 private; East region- 6 sites, 5 public and 1 private; West region- 3 sites, 2 public and 1 private) were selected through a systematic process. Out of these sites, four sites were located in the states where RVV was introduced in April 2017 under phase 1. At all sites the RVVs were available in private market during the study period, even prior to the introduction in NIP.

### Case definition, case selection and data collection

The children aged 2–23 months admitted to these hospitals with diagnosis of intussusception were eligible. All the age-eligible patients admitted were screened to identify the suspected cases (any of the diagnoses: intussusception, acute or subacute intestinal obstruction, acute abdomen, abdominal pain, abdominal distension, and blood in stool with vomiting). These suspected cases were tracked till final diagnosis and all confirmed intussusception cases were recruited after written informed consent from parent or legally authorised representative. A log of screened, suspected and confirmed cases was maintained. The data on socio-demography, feeding and immunization (from immunization card), clinical features, investigation findings, hospital course, treatment and outcome were collected using common case record form (CRF). The symptoms were captured as recorded in the case sheets and reported by the parents. An independent Case Adjudication Committee (CAC) comprised of a paediatrician, paediatric surgeon, and radiologist reviewed the CRFs and investigations to assign the diagnostic certainty levels, according to Brighton Collaboration case definition (BCCD) [21].

### Quality assurance

Multilevel quality assurance and data quality-checking processes were put in place to ascertain protocol adherence, rigor and completion of surveillance at all sites. A data team reviewed all the CRFs and data-related query was resolved at the earliest. Each site was visited by external experts (Technical Advisory Group, TAG) to assess the case surveillance and tracking, consent, and data extraction quality and completeness in the CRFs. The TAG members checked CRFs completeness and quality for few randomly identified cases with the case records. Subsequently members from the data team visited the study sites and checked the admissions for the study period from the medical records section using diagnoses and International Classification of Diseases (ICD) codes (ICD-9/10, codes listed in Supplementary Table 1), to identify any missed cases.

### Data management and analysis

Double data entry was done for the CRFs using a customised data entry platform. The matched and verified data were stored in the server with authorised access and daily backup. The standard of living index (SLI), representing the socioeconomic status was estimated using the scores for household assets ownership, with reference to the National Family and Health Survey for India [22]. The SLI was categorised into high, medium and low categories. The cases were categorised into levels 1 to 3 based on the BCCD criteria by the CAC [21]. In view of the number of sites, for representation we grouped the sites into four regions, North (5 sites), South (5 sites), East (6 sites) and West (3 sites) regions. The parameters (sociodemographic, feeding, clinical, intervention and outcomes were compared between the regions to detect variations. The intussusception classical triad includes three features; abdominal pain, vomiting and blood in stools. We considered intussusception modified triad with abdominal pain, vomiting and rectal bleeding, detected either as blood in stool or blood on per-rectal examination. The descriptive analysis findings expressed the outputs as proportions, means and standard deviations, or median and interquartile range (IQR), as appropriate. The values between regions and groups were compared for statistical significance using Chi-Square or Fisher’s exact tests for the proportions and Mann–Whitney U or Kruskal-Wallis tests for the medians depending on the skewness, sample and number of groups. The missing data were excluded from analysis. The statistical significance was considered if *p* < 0.05. The statistical analysis was performed using STATA version 15.0 (StataCorp LLC, Texas, USA). In the Indian context, there is no definite population catchment area for hospitals and no definite referral chain, which makes estimation of the intussusception incidence difficult. For comparison of the data across sites and intussusception time trend, in addition to case load (absolute number of cases), we attempted deriving the intussusception case rate per 1000 paediatric hospitalisations at the hospitals. On review, while the admission numbers in the paediatric medicine wards varied widely, the admission numbers in the paediatric surgery wards were relatively stable and the intussusception cases were primarily managed in the paediatric surgery wards. Thus we estimated the intussusception case rate per 1000 paediatric surgery admissions at these hospitals for comparison and trend analysis. For the four sites in three states (Odisha, Andhra Pradesh and Haryana), where RVV (monovalent Rotavac™, 3 doses at 6, 10, 14 weeks age) was introduced under NIP (in April 2017), the data for the post-introduction period (April to September 2017, 6 months) was compared with the pre-introduction periods (first: October 2016 to March 2017, immediate 6 months pre-introduction period and second: April 2016 to September 2016, calendar matched 6 months during the previous year) to document the risk ratio (95% confidence interval, CI). The detailed methodology of the site selection and study implementation has been published as protocol [23].

### Ethical issues

Informed written consent was obtained for all the eligible cases before recruitment and data collection. Confidentiality in data handling was maintained. The study protocol was reviewed and approved by the ethics committees of all participating institutes.

## Results

**Fig. 1:**
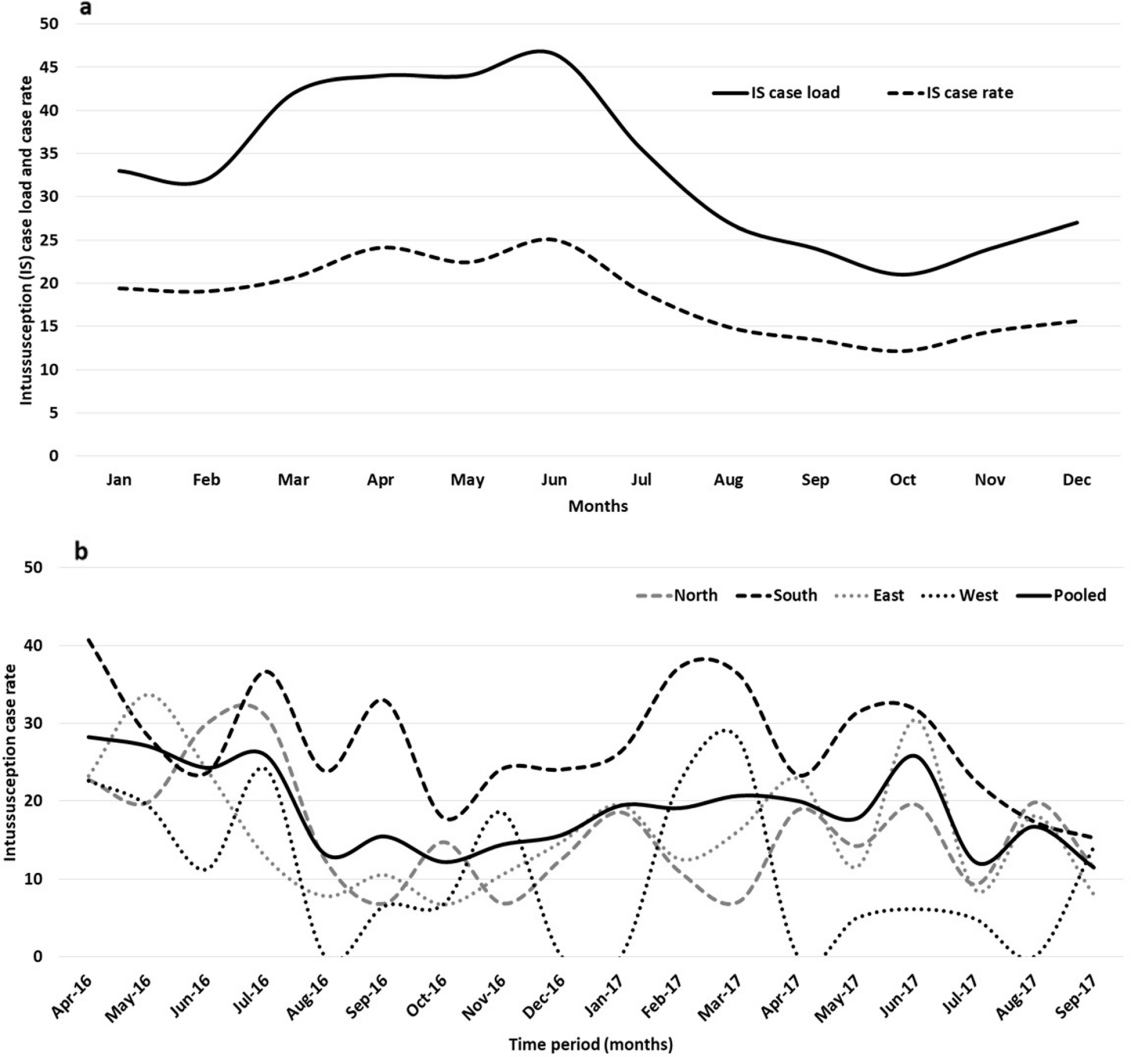
The seasonal distribution of pooled intussusception case load and case rate during the calendar months (**a**) and regional intussusception case rates during the study period (**b**). Note: Intussusception case rate: Intussusception cases per 1000 paediatric surgery admissions at the hospitals

Between April 2016 and September 2017, out of the 182,824 children (including 32,910 paediatric surgery admissions) admitted to the network hospitals, 1203 suspected intussusception cases were identified and 621 eligible children were recruited (Supplementary Figure 2). More cases were recruited from Southern region (42.2%; 262/621) followed by East (30.6%; 190/621), North (21.9%; 136/621) and West (5.3%; 33/621) regions. Past history of intussusception was present in 24 (3.8%) cases. Table 1 shows the sociodemographic characteristics of children with intussusception. A male predominance (male-female ratio: 1.9:1) was consistent across all regions. The median age at presentation was 8 months (IQR 5, 13 months) (Supplementary Figure 3). Three quarters of the cases were infants with equal share from the age bands of 2–6 months (37.2%) and 7–12 months (37.7%) (*p* < 0.01). Children aged 4–7 months contributed to 40.0% of the total cases. The pooled intussusception case rate per 1000 paediatric surgery admission was 18.4 (IQR 14.7, 23.4). The intussusception case rate per 1000 paediatric surgery admission was highest for South (25.3, IQR 23.4, 32.7) followed by North (14.5, IQR 11, 19.7), East (13.8, IQR 10.5, 22) and West (6.6, IQR 1.2, 19.2) regions respectively. The Fig. 1 shows monthly trends of pooled intussusception case load and case rates per 1000 paediatric surgery admission (Fig. 1a) and the regional case rates per 1000 paediatric surgery admission (Fig. 1b). More cases (*n* = 311, 50.0%) were seen during March to June months, in the summer season (Fig. 1). While 42.8% of the patients were resident of the same district where the hospital was based, 41.9% of the patients presented directly to the hospital. Among the children aged > 6 months, 23.2% were exclusively breastfed for 6 months and the median duration of breastfeeding was 4 months. Mixed feeding was initiated before 6 months of age in 53.9% children. The median weaning age was 6 months and 61.0% received rice based food. Ragi (finger millet) was given to 22.8% children, only in the South region. The interval between weaning and intussusception was significantly shorter for ragi (median 1 month, IQR 0–4.2 months) than rice (median 4 months, IQR 1–9 months) and wheat (median 3 months, IQR 1–7 months) based food (Table 1) (*p* < 0.01). On analysis for the South region only (where ragi based weaning practice was observed), the median interval between weaning and intussusception for ragi based food was significantly shorter (median 1 month, IQR 0–4 months) than rice (median 5 months, IQR 2–9 months) and wheat (median 3 months, IQR 2–8 months) based food (*p* < 0.01).

**Table 1:** The sociodemographic parameters and dietary practices of children with intussusception in India

Variable Category		North (*n* = 136)	South (*n* = 262)	East (*n* = 190)	West (*n* = 33)	Total (*n* = 621)	*P* value
Age (in months)	2–6, n (%)	55 (40.4)	88 (33.6)	78 (41.1)	10 (30.3)	231 (37.2)	< 0.01
	7–12, n (%)	57 (41.9)	92 (35.1)	73 (38.4)	12 (36.4)	234 (37.7)	< 0.01
	13–18, n (%)	16 (11.8)	40 (15.3)	25 (13.2)	3 (9.1)	84 (13.5)	< 0.01
	19–23, n (%)	8 (5.9)	42 (16.0)	14 (7.4)	8 (24.2)	72 (11.6)	< 0.01
Gender	Male, n (%)	86 (63.2)	170 (64.9)	128 (67.4)	24 (72.7)	408 (65.7)	< 0.01
	Female, n (%)	50 (36.8)	92 (35.1)	62 (32.6)	8 (24.2)	212 (34.1)	< 0.01
	Other, n (%)	0 (0.0)	0 (0.0)	0 (0.0)	1 (3.0)	1 (0.2)	–
Family	Nuclear, n (%)	89 (65.4)	171 (65.2)	65 (34.2)	18 (54.5)	343 (55.2)	0.06
	Joint/extended, n (%)	47 (34.6)	91 (34.7)	125 (65.8)	15 (45.5)	278 (44.8)	0.06
Religion	Hindu, n (%)	48 (35.3)	179 (68.3)	136 (71.6)	22 (66.6)	385 (62.0)	< 0.01
	Muslim, n (%)	88 (64.7)	41 (15.6)	52 (27.3)	11 (33.3)	192 (30.9)	< 0.01
	Christian, n (%)	0 (0.0)	42 (16.0)	1 (0.5)	0 (0.0)	43 (7.0)	–
SLI status^a^	High, n (%)	56 (41.2)	93 (35.5)	19 (10.0)	6 (18.2)	174 (28.0)	< 0.01
	Medium, n (%)	33 (24.3)	124 (47.3)	65 (34.2)	6 (18.2)	228 (36.7)	< 0.01
	Low, n (%)	47 (34.5)	45 (17.2)	106 (55.8)	21 (63.6)	219 (35.3)	< 0.01
Place of residence	Same district, n (%)	49 (36.0)	162 (61.8)	35 (18.4)	20 (60.6)	266 (42.8)	< 0.01
	Other districts^b^, n (%)	74 (54.4)	86 (32.8)	151 (79.5)	13 (39.4)	324 (52.2)	< 0.01
	Outside state, n (%)	13 (9.6)	14 (5.3)	4 (2.1)	0 (0.0)	31 (5.0)	< 0.01
Referral status	Primary^c^, n (%)	91 (66.9)	64 (24.4)	87 (45.8)	18 (54.6)	260 (41.9)	< 0.01
	Referred^d^, n (%)	45 (33.1)	198 (75.6)	103 (54.2)	15 (45.5)	361 (58.1)	< 0.01
Feeding practices	Exclusively breastfed for 6 months^e^, n/N (%)	3/26 (11.5)	16/52 (30.7)	18/85 (21.2)	1/1 (100)	38/164 (23.2)	< 0.01
	Mixed food before 6 months age^e^, n/N (%)	25/78 (32.0)	130/197 (66.0)	46/100 (46.0)	12/20 (60.0)	213/395 (53.9)	0.08
	Exclusive breastfeeding period (in months), median (IQR)	5 (4–6)	4 (2–5)	4 (3–5)	5 (3.5–5)	4 (3–7)	–
	Mixed food initiation age in months, median (IQR)	6 (5–8)	4 (2–6)	5 (3–6)	5 (4–6)	5 (3–7)	–
	Weaning age in months, median (IQR)	7 (6–8.2)	6 (4–6)	6 (5–7)	6 (5–7)	6 (5–7)	–
Weaning food type	Rice, n (%)	48 (68.6)	116 (53.0)	80 (88.9)	5 (18.5)	249 (61.0)	< 0.01
	Wheat n (%)	12 (17.1)	5 (23.0)	3 (3.3)	11 (40.7)	31 (8.0)	< 0.01
	Ragi based^f^, n (%)	0 (0.0)	50 (22.8)	0 (0.0)	0 (0.0)	50 (12.0)	–
	Other, n (%)	4 (5.7)	7 (3.2)	3 (3.3)	4 (14.8)	18 (4.0)	0.08
	Mixed n (%)	6 (8.6)	41 (18.7)	4 (4.4)	7 (25.9)	58 (14.0)	0.05
Weaning initiation to illness onset interval (in months)	Rice based, median (IQR)	3 (0–6)	3 (1–9)	4 (1–9)	4 (2–11.5)	4 (1–9)	0.08
	Wheat based, median (IQR)	3 (2.5–7)	2.5 (1.2–5.2)	0 (0–1)	3 (2–7)	3 (1–7)	0.05
	Ragi^f^, median (IQR)	–	1 (0–4.2)	–	–	1 (0–4.2)	–
	Other food, median (IQR)	4 (1.5–6)	1 (0–3)	0 (0–1)	9.5 (5.2–13)	2.5 (0.7–5.5)	0.09
	Multiple food, median (IQR)	7 (2.5–9)	6 (2.7–12)	6 (3–12)	6 (3–12)	6 (3–12)	0.1
	Any food, median (IQR)	3 (1–7)	3 (1–8)	3.5 (1–8.2)	3 (1–9)	3.5 (1–8.2)	0.1

Notes:

Mixed food: Food used for weaning included at least two of the ingredients- rice, wheat, lentil and vegetables & fruits

^a^SLI: Standard of living index, was estimated using the household assets ownership (22)

^b^The residence of the patient was in other district in the state where the study hospital was based

^c^The patient presented to the study hospital as the first institute

^d^The patient was referred to the study hospital from another hospital

^e^The denominators (N) indicate valid responses received for the parameters

^f^Ragi: finger millet

The p values for some parameters could not be estimated due to small or no value in at least two groups

The pooled median interval between the onset to hospital admission was 2 days (IQR 1, 3), which varied across the regions; North- 2 days (IQR 1, 3), South- 1 day (IQR 1, 3), East- 2 days (IQR 1, 3) and West- 2 days (IQR 1, 2) (*p* = 0.07). Table 2 summarises the clinicopathological parameters and management for the recruited children. Abdominal pain or excessive crying (82.6%) was the most common symptom followed by vomiting (72.6%) and blood in stool (58.1%), as reported by the parents. More children had abdominal pain or excessive crying in North (84.6%) and East (72.6%) regions and blood in stool in the East region (73.2%). The classical triad (abdominal pain, vomiting and blood in stool) was observed in 34.8% cases, with 24.4 and 45.8% in the South and in East regions, respectively (*p* < 0.01). When blood on per-rectal examination was included, the modified intussusception triad (abdominal pain, vomiting and blood in stool or blood on per-rectal examination) was present in 59.9% cases; 47.7% in South to 75.3% in East regions (*p* < 0.01).

**Table 2:** The clinicopathological characteristics and management of the children with intussusception in India

Variable Category		North (*n* = 136)	South (*n* = 262)	East (*n* = 190)	West (*n* = 33)	Total (*n* = 621)	*P* value
Symptoms, n (%)	Vomiting^a^	100 (73.5)	187 (71.4)	137 (72.1)	27 (81.8)	451 (72.6)	0.64
	Bilious vomiting^b^	21 (15.4)	26 (9.9)	34 (17.9)	9 (27.3)	90 (14.5)	0.02
	Abdominal pain	102 (75.0)	118 (45.0)	138 (72.6)	10 (30.3)	368 (59.3)	< 0.01
	Excessive crying	49 (36.0)	172 (65.7)	109 (57.4)	17 (51.5)	347 (55.9)	< 0.01
	Abdominal distension	34 (25.0)	29 (11.1)	69 (36.3)	3 (9.1)	135 (21.7)	< 0.01
	Rectal bleeding	81 (59.6)	123 (47.0)	139 (73.2)	18 (54.6)	361 (58.1)	< 0.01
	Diarrhoea	23 (16.9)	69 (26.3)	37 (19.5)	9 (27.3)	138 (22.2)	0.11
	Constipation	18 (13.2)	43 (16.4)	12 (6.3)	3 (9.1)	76 (12.2)	0.01
	Fever	34 (25.0)	64 (24.4)	31 (16.3)	13 (39.4)	142 (22.9)	0.02
	Lethargy	2 (1.5)	16 (6.1)	16 (8.4)	2 (6.1)	36 (5.8)	0.07
	Altered sensorium	1 (0.7)	0 (0.0)	0 (0.0)	0 (0.0)	1 (0.2)	–
	Classical triad^c^	52 (38.2)	64 (24.4)	87 (45.8)	13 (39.4)	216 (34.8)	< 0.01
Signs, n (%)	Pallor	11 (8.1)	14 (5.3)	18 (9.5)	3 (9.1)	46 (7.4)	0.39
	Dehydration	7 (5.2)	21 (8.0)	43 (22.6)	2 (6.1)	73 (11.8)	< 0.01
	Fever	4 (2.9)	23 (8.8)	23 (12.1)	5 (15.2)	55 (8.9)	0.02
	Lethargy	11 (8.1)	14 (5.3)	20 (10.5)	3 (9.1)	48 (7.7)	0.23
	Abdominal distension	22 (16.2)	25 (9.5)	62 (32.6)	3 (9.1)	112 (18.0)	< 0.01
	Abdominal tenderness	13 (9.6)	26 (9.9)	26 (13.7)	5 (15.2)	70 (11.3)	0.48
	Abdominal mass	13 (9.6)	91 (34.7)	24 (12.6)	4 (12.1)	132 (21.3)	< 0.01
	Bowel sound (absent/ abnormal)	11 (8.1)	10 (3.8)	43 (22.6)	2 (6.1)	66 (10.6)	< 0.01
	Rectal prolapse	15 (11.0)	2 (0.8)	1 (0.5)	0 (0.0)	18 (2.9)	< 0.01
	Rectal mass	3 (2.2)	4 (1.5)	11 (5.8)	0 (0.0)	18 (2.9)	0.04
	Blood on PR examination	19 (14.0)	31 (11.8)	94 (49.5)	19 (57.6)	163 (26.3)	< 0.01
	Modified triad^d^	83 (61.0)	125 (47.7)	143 (75.3)	21 (63.6)	372 (59.9)	< 0.01
Diagnosis method, n (%)	Ultrasound	121 (89.0)	262 (100)	179 (94.2)	30 (90.9)	592 (95.3)	< 0.01
	CT scan	2 (1.5)	0 (0.0)	0 (0.0)	0 (0.0)	2 (0.3)	–
	Barium enema	1 (0.7)	0 (0.0)	1 (0.5)	1 (3.0)	3 (0.5)	–
	Surgery	12 (8.8)	0 (0.0)	10 (5.3)	2 (6.1)	24 (3.9)	< 0.01
Intussusception location, n (%)	Colo-colic	3 (2.2)	10 (3.8)	5 (2.6)	3 (9.1)	21 (3.4)	< 0.01
	Ileo-colo-colic	8 (5.9)	7 (2.7)	4 (2.1)	2 (6.1)	21 (3.4)	< 0.01
	Ileo-ileal	11 (8.1)	3 (1.2)	10 (5.3)	6 (18.2)	30 (4.8)	< 0.01
	Ileo-ileo-colic	4 (2.9)	5 (1.9)	6 (3.2)	0 (0.0)	15 (2.4)	< 0.01
	Ileocolic	109 (80.2)	234 (89.3)	163 (85.8)	20 (60.1)	526 (84.7)	< 0.01
	Jejuno-jejunum	0 (0.0)	0 (0.0)	1 (0.9)	1 (3.0)	2 (0.3)	–
	> 1 location^e^	1 (0.7)	3 (1.2)	1(0.5)	1 (3.0)	6 (1.0)	–
Pathological lead point, n (%)	Lymph node	41 (30.1)	19 (7.2)	3 (1.6)	15 (45.4)	78 (12.6)	< 0.01
	Appendix	0 (0.0)	0 (0.0)	2 (1.0)	1 (3.0)	3 (0.4)	–
	Payer’s patch	1 (0.7)	1 (0.3)	1 (0.5)	0 (0.0)	3 (0.4)	–
	Polyp	1 (0.7)	0 (0.0)	0 (0.0)	1 (3.0)	2 (0.3)	–
	Others^f^	2 (1.4)	0 (0.0)	3 (1.6)	0 (0.0)	5 (0.8)	–
	Any lead point	45 (33.0)	20 (7.6)	9 (4.7)	17 (51.5)	91 (14.6)	< 0.01
Treatment modality, n (%)	Reduction	56 (41.2)	216 (82.4)	20 (10.5)	14 (42.4)	306 (49.3)	< 0.01
	Surgery	68 (50.0)	30 (11.5)	135 (71.1)	12 (36.4)	245 (39.5)	< 0.01
	Spontaneously resolved^g^	12 (8.8)	16 (6.1)	34 (17.9)	7 (21.2)	69 (11.1)	< 0.01
	None^h^	0 (0.0)	0 (0.0)	1 (0.5)	0 (0.0)	1 (0.2)	–
Surgery type, n (%)	No resection^i^	41 (60.3)	25 (83.3)	106 (78.6)	9 (75)	181 (73.9)	< 0.01
	With resection^i^	27 (39.7)	5 (16.7)	29 (21.5)	3 (25.0)	64 (26.1)	< 0.01
Outcome, n (%)	Discharged	129 (94.9)	262 (100)	181 (95.3)	31 (93.9)	603 (97.1)	< 0.01
	Referred	0 (0.0)	0 (0.0)	1 (0.5)	2 (6.1)	3 (0.5)	–
	LAMA^j^	3 (2.2)	0 (0.0)	1 (0.5)	0 (0.0)	4 (0.6)	–
	Died	4 (2.9)	0 (0.0)	7 (3.7)	0 (0.0)	11 (1.8)	–
Brighton Collaboration Criteria level, n (%)	Level 1	125 (91.9)	246 (93.9)	156 (82.1)	26 (78.8)	553 (89.1)	< 0.01
	Level 2	5 (3.7)	15 (5.7)	24 (12.6)	3 (9.1)	47 (7.6)	< 0.01
	Level 3	0 (0.0)	0 (0.0)	0 (0.0)	0 (0.0)	0 (0.0)	–
	No level	6 (4.4)	1 (0.4)	10 (5.3)	4 (12.1)	21 (3.4)	< 0.01
Illness in last 4 weeks, n (%)	Diarrhoea	10 (7.4)	55 (21.0)	11 (5.8)	4 (12.1)	80 (12.9)	0.04
	ARI	17 (12.5)	104 (39.7)	21 (11.1)	5 (15.2)	147 (23.7)	0.04

Notes: *PR* Per-rectal, *ARI* Acute respiratory illnesses

^a^Includes all vomiting including bilious vomiting

^b^Includes only bilious vomiting

^c^Classical triad includes abdominal pain, vomiting and blood in stool

^d^Modified triad includes abdominal pain, vomiting and rectal bleeding (detected either as blood in stool or blood on per-rectal examination)

^e^The location of intussusception at more than one site; such as Ileo-colic + Colo-colic, Ileo-colic + Ileo-colo-colic, Ileo-colic + Ileo-ileo-colic, Ileo-colic + Ileo-ileal

^f^Others include cyst, Meckel’s diverticulum, incisional hernia, intraluminal growth and malrotation

^g^Spontaneously resolved: The patient was managed with IV fluids and nil orally, not required any additional intervention

^h^ None- No definite treatment could be provided, as the patient(s) was referred or died before any definite treatment

^i^ The denominators used for percentage estimation include patients undergone surgery only

^j^LAMA: Left against medical advice

The p values for some parameters could not be estimated due to no value in at least two groups

Most of the cases (95.3%) were diagnosed by ultrasound. Ileocolic (84.7%) was the most common site of intussusception. A pathological lead point (PLP) was documented in 91 (14.6%) cases and lymph nodes or Payer’s patch were the commonest (13.0%) and few had appendix or polyps (Table 2). While 49.3% cases were reduced, 39.5% underwent surgery and 11.1% resolved spontaneously. More children in East region (71.1%) underwent surgery followed by North region (50.0%) (*p* < 0.01). Among those who underwent surgery, 26.1% children required bowel resection, and 39.5% were from the North region. The onset-admission interval was longer for the cases who underwent surgery (median 2 days; IQR 1, 3) than reduction (median 1 day; IQR 1, 3) (*p* < 0.01) (Supplementary Table 2). Among the children who presented on the day of onset, 62.3% were managed by reduction (28.9% required surgery), which declined to 35.1% when they presented after 3 days (48.3% required surgery) (*p* < 0.01) (Supplementary Table 2). Seven hospitals (East-1, North-1, and South-1) were conducting only surgical management. At the hospitals with both facilities (reduction and surgery), 31.0% (127/404) children underwent surgery for indications: failed reduction (32.2%), late presentation (> 3 days since onset, 28.3%) and associated complications (39.3%) (*p* < 0.01). Most of the cases (97.1%) recovered and were discharged. Eleven cases (North-4 and East-7) died of post-surgery sepsis, shock and multiorgan failure. The median hospitalisation period was 3 days (IQR 2, 6 days) ranging from 2 to 5 days (South: 2 days, IQR 2, 3 days; North: 3 days, IQR 1, 7 days; West: 4 days, IQR 2, 6 days; and East: 5 days, IQR 3, 8 days; *p* < 0.01). The median hospital stay periods for children who underwent surgery, reduction and spontaneously resolved were 7 days (IQR 5, 9 days), 2 days (IQR 1, 2 days) and 3 days (IQR 2, 5 days), respectively (*p* < 0.01) (Supplementary Table 2). CAC assigned 89.1% (553/621) and 7.6% cases as Level 1 and 2 respectively according to the BCCD. Diarrhoea and acute respiratory illnesses (ARIs) within 4 weeks prior to intussusception was reported in 12.9 and 23.7% children respectively. More children from South region had history of diarrhoea (21%) and ARIs (39.7%) than other regions (*p* < 0.05).

**Fig. 2:**
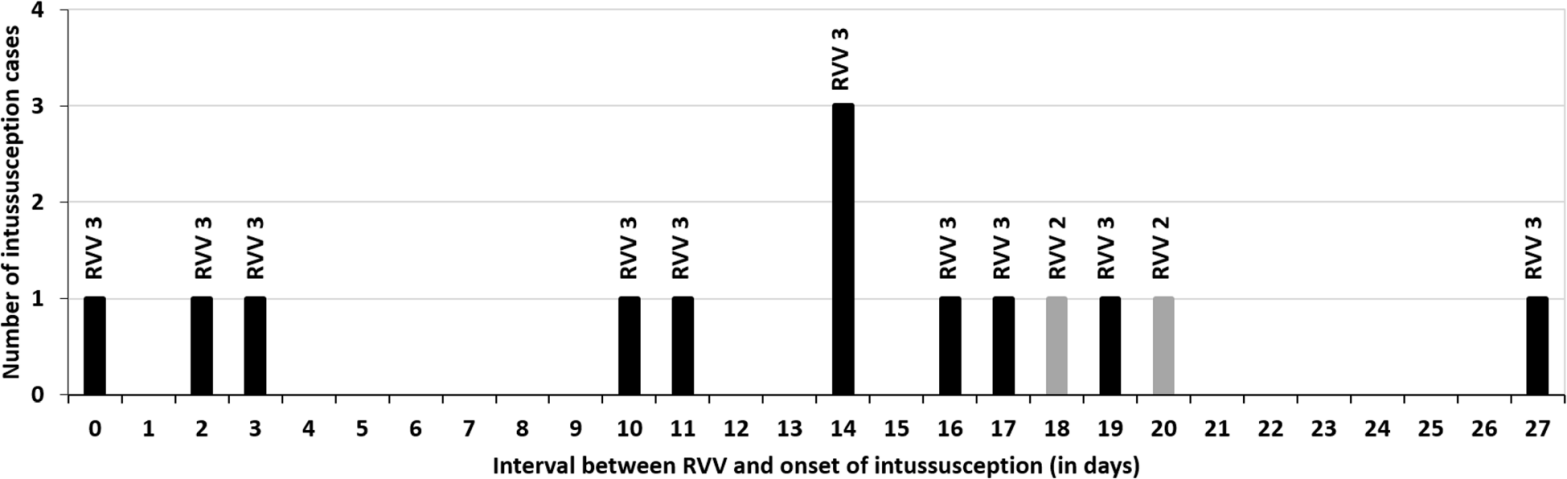
Interval between last rotavirus vaccination and onset of intussusception within 28 days in the children. Note: RVV: Rotavirus vaccine

Vaccination information was available for 487 (78.4%) children and 391 (80.2%) of those with vaccination information had no RVV exposure. Out of 96 (15.4%) children who received any RVV (RVV-1, *n* = 96; RVV-2, *n* = 88; and RVV-3, *n* = 65), 12 children received RVV in the 1–21 days preceding onset of intussusception and most (*n* = 10) after RVV-3 (median age 3.8 months, IQR 3.6, 4.2 months) (Supplementary Table 3). As shown in Fig. 2, only two cases occurred during 1–7 days after the third dose RVV and one case occurred on the vaccination day. At the four sites from three states where RVV (Rotavac™) was introduced, no increase in either intussusception case load (RR = 0.44; 95% CI 0.22, 1.18) or case rate per 1000 paediatric surgery admission (RR = 0.5; 95% CI 0.3, 1.2) during the post-RVV introduction period were observed (Table 3).

**Table 3:** The intussusception cases and case ratios during post-rotavirus vaccine introduction period compared to pre-introduction periods

	Post-RVV period (Apr- Sep 2017)		Pre-RVV period (Oct 2016 - Mar 2017)				Pre-RVV period (Apr- Sep 2016)			
Region (number of sites)^a^	No of cases	IS case rate	No of cases	IS case ratio	RR (95% CI) for cases	RR (95% CI) for IS case rate	No of cases	IS ratio	RR (95% CI) for cases	RR (95% CI) for IS case rate
East (*n* = 2)^a^	20	20.4	29	29.9	0.69 (0.33–1.2)	0.67 (0.3–1.28)	45	34.6	0.44 (0.2–1.15)	0.58 (0.32–1.2)
North (*n* = 1)^a^	0	0	2	27.4	–	–	3	37.5	–	–
South (*n* = 1)^a^	6	13.3	8	20.4	0.75 (0.45–1.24)	0.65 (0.48–1.23)	11	26.4	0.55 (0.28–1.2)	0.51 (0.3–1.22)
Pooled (*n* = 4)^a^	26	16.3	39	27.2	0.67 (0.34–1.19)	0.6 (0.3–1.18)	59	32.8	0.44 (0.22–1.18)	0.5 (0.3–1.2)

Note: *IS* Intussusception, *Intussusception (IS) case rate* Intussusception cases per 1000 paediatric surgery admission, *RVV* Rotavirus vaccine, *RR* risk ratio with 95% confidence interval (CI), *RVV*: Rotavirus vaccine

The comparison includes the data from four study sites in three states

^a^Numbers in the bracket with the regions indicate the numbers of study sites per region where RVV was introduced

Post-RVV period: April 2017 to September 2017 (6 months)

Pre-RVV period: Comparison has been done for two pre-intervention periods of 6 months each; October 2016 to March 2017- the immediate pre-RVV introduction period and April 2016 to September 2016- the calendar months comparable period during the previous year

## Discussion

We observed regional variances in the intussusception case load and case rate per 1000 paediatric surgery admission, high in the South region and low in the West region. Higher number of cases were observed during March to June months, which was comparable to other reports from India [14, 15, 17, 24, 25]. The male predominance (65.7%), median age at 8 months and high case load at 4–7 months of age (40.0%) were similar to other reports from India and globally (proportion of male: 63.0–77.2%; median age: 5–8 months and high case load age: 4–9 months) [14, 15, 17, 18, 21, 24, 26, 27]. About 37.0% of cases occurred during 2nd to 6th month of age, the usual age for administration of RVV. The children came from all socioeconomic categories and in all the regions. The proportion of exclusively breastfed children (< 6 months) was lower than national average (52.1%, 2015–16) [22]. The children weaned with ragi food (only in South region) had intussusception earlier (median 1 month) than those weaned with wheat (median 3 months) and rice (median 4 months) based food. A shorter exclusive breastfeeding duration with mixed feeding and some weaning foods may have some role in intussusception occurrence. Exposure to foreign proteins and enteric infections are potential risk factors for intussusception, but we couldn’t find any report on association with any specific diet. There were significant variations for several sociodemographic and dietary practice parameters across the regions (Table 1). The classical triad was documented in 34.8% cases, which was higher than reports from India (19.0%) and South Korea (7.6%), but lower than Tanzania (42.5%) [15, 24, 28]. The regional variation in the classical triad appeared to parallel with the interval between illness onset and hospitalization. Ultrasound was the commonest (95.3%) mode of diagnosis, as reported (72.0–100%) from India and other countries [15, 18, 28–30]. Ileocolic was the most common location, similar to reports (68.0–79.0%) from India and globally [3, 7, 8, 14]. PLPs observed in this study was higher than reported in studies (8.0–9.0%) from India and Tanzania with appendix and lymph nodes as the common [31, 32]. The cases who presented early were more frequently managed non-surgically with shorter stay (2–3 days) compared to those with surgical intervention (7 days). There were significant variations in the clinical features among the children across the regions, which may be due to the interval for presentation or clinical practices followed at the hospitals (Table 2). Death was observed in 1.7% cases, which was comparable to reports from India (1.0%) and other countries from Asia (0.25–6.0%), Latin America (1.0–5.0%), but lower than the African countries (2.0–25.0%) [17, 18, 24, 28, 29, 31–33]. Level 1 BCCD was met by 89.1% of the cases, similar to other reports from India [5, 7, 14]. Failed documentation of reduction prevented some cases meeting the BCCD level 1 certainty.

From the limited period of post-RVV (Rotavac™) introduction at four sites (from three states), no increase in the intussusception case load and case rate per 1000 paediatric surgery admission were observed. Intussusception within 1–21 days was observed mostly after the third RVV dose, which also overlaps with the age of natural occurrence. Under the NIP, RVV is given at 6, 10, 14 weeks of age and allowed before 12 months of age [34]. A delay in RVV administration may coincide with the high case load age of natural intussusception (4–7 months age), which may make the interpretation of association with the vaccine difficult. In routine practice, documentation of vaccine exposure should improve and appropriate guidance to parents be given regarding the subsequent RVV vaccination. Monitoring of intussusception risk is recommended for countries as part of the RVV introduction program [35, 36]. In the absence of any specific population denominator for tertiary care hospitals, estimation of incidence in Indian context is difficult. The intussusception case rate per 1000 paediatric surgery admissions may be a proxy indicator for monitoring and comparison across the sites and regions with RVV introduction.

Early suspicion, case detection and referral to appropriate hospital are critical for minimizing the surgical interventions and favourable outcomes. Efforts are needed to equip and enable the public health facilities for non-surgical management in children, though this depends on the timing of the presentation to hospital.

The epidemiology, clinical presentation and management of intussusception in children across the regions may serve as baseline for future studies. The regional representation and mix of private- and public-sector hospitals were the advantages for this surveillance.

This study had some limitations. In absence of definite catchment population and referral pattern, population-based incidence or case rate estimation was not possible. The etiologies and risk factors for intussusception were not studied.

## Conclusion

To conclude, the intussusception cases were seen all across the country and the majority of the cases occurred during the first year of life. The high case load age (4–7 months) for intussusception in children coincided with the age of RVV third dose. Some variations in case loads across the different regions and seasonality were observed. The potential role of diet exposure, weaning practices and food like ragi in intussusception needs further evaluation. No increased occurrence of intussusception cases was observed during the limited RVV post-introduction period. Early case detection, prompt referral and appropriate management are needed to avoid surgical intervention and complications. Immunization exposure must be documented to assess the vaccine associated risk. In absence of population-based incidence or case rate, the intussusception case rate per 1000 paediatric surgery admission may be used for inter-regional comparison and trend monitoring.

## Supplementary information

**Additional file 1.** Supplementary document 1- Supplementary Figure 1: The study sites and their locations according to the regions. Supplementary document 2- Supplementary Table 1: The International Classification of Diseases (ICD) codes for review of the cases from medical records. Supplementary document 3- Supplementary Figure 2: The flow chart for case screening and recruitment. Supplementary document 4- Supplementary Figure 3: Age distribution of children with intussusception in India (region wise and pooled). Supplementary document 5- Supplementary Table 2: The mode of treatment according to the interval between onset, admission and intervention for the children with intussusception. Supplementary document 6- Supplementary Table 3: The intussusception cases during risk periods after rotavirus vaccine exposure.

## Data Availability

All data is available with the investigators and can be provided by the corresponding author upon reasonable request.

## Abbreviations

CRF: Case record form
CAC: Case Adjudication Committee
ICD: International Classification of Diseases
IQR: Interquartile range
TAG: Technical Advisory Group
WHO: World Health Organization
RVV: Rotavirus vaccine
SLI: Standard of living index
NIP: National immunization programmes.
