# Supplementary material for: Prospective surveillance for intussusception in Indian children aged under two years at nineteen tertiary care hospitals
Source: BMC Pediatr. 2020 Sep 1;20:413. doi: 10.1186/s12887-020-02293-5 (PMC7461288; doi:10.1186/s12887-020-02293-5)
Supplement: Supplementary file 1 — Additional file 1. Supplementary document 1- Supplementary Figure 1: The study sites and their locations according to the regions. Supplementary document 2- Supplementary Table 1: The International Classification of Diseases (ICD) codes for review of the cases from medical records. Supplementary document 3- Supplementary Figure 2: The flow chart for case screening and recruitment. Supplementary document 4- Supplementary Figure 3: Age distribution of children with intussusception in India (region wise and pooled). Supplementary document 5- Supplementary Table 2: The mode of treatment according to the interval between onset, admission and intervention for the children with intussusception. Supplementary document 6- Supplementary Table 3: The intussusception cases during risk periods after rotavirus vaccine exposure. [file 12887_2020_2293_MOESM1_ESM.docx]

**Prospective Surveillance for Intussusception in Indian Children Aged Under Two Years at Nineteen Tertiary Care Hospitals**

**Supplementary Documents**

**Index**

1. Supplementary Figure 1: The study sites and their locations according to the regions
2. Supplementary Table 1: The International Classification of Diseases (ICD) codes for review of the cases from medical records
3. Supplementary Figure 2: The flow chart for case screening and recruitment
4. Supplementary Figure 3: Age distribution of children with intussusception in India (region wise and pooled)
5. Supplementary Table 2: The mode of treatment according to the interval between onset, admission and intervention for the children with intussusception
6. Supplementary Table 3: The intussusception cases during risk periods after rotavirus vaccine exposure
7. Supplementary Figure 1: The study sites and their locations according to the regions


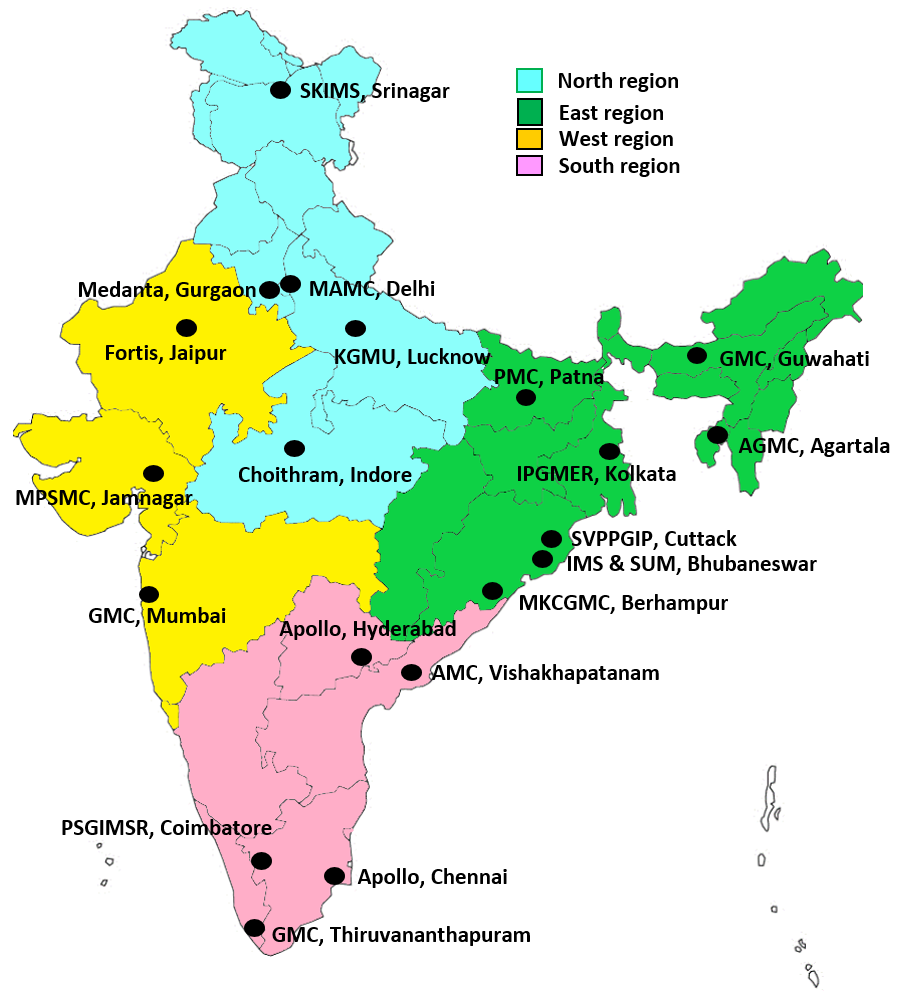


*Note: The map has been prepared for use in the research project. The corresponding author is the creator of the image.*

1. Supplementary Table 1: The International Classification of Diseases (ICD) codes for review of the cases from medical records

| Clinical conditions considered as suspected cases | Codes | |
| --- | --- | --- |
|  | ICD 10 | ICD 9 |
| Intussusception | K56.1 | 560.0 |
| Volvulus | K56.2 | 560.2 |
| Gallstone ileus | K56.3 | 560.31 |
| Other impaction of intestine | K56.4 | 560.30 |
| Intestinal adhesions with obstruction | K56.5 | 560.81 |
| Other and unspecified intestinal obstruction | K56.6 | 560.9 |
| Ileus, unspecified | K56.7 | 560.1 |
| Paralytic ileus | K56.0 |  |

*Note: ICD: International Classification of Diseases*

1. Supplementary Figure 2: The flow chart for case screening and recruitment


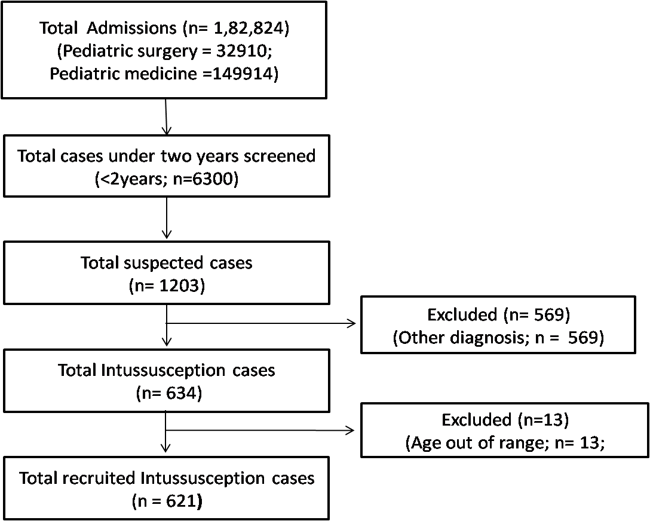


1. Supplementary Figure 3: Age distribution of children with intussusception in India (region wise and pooled)


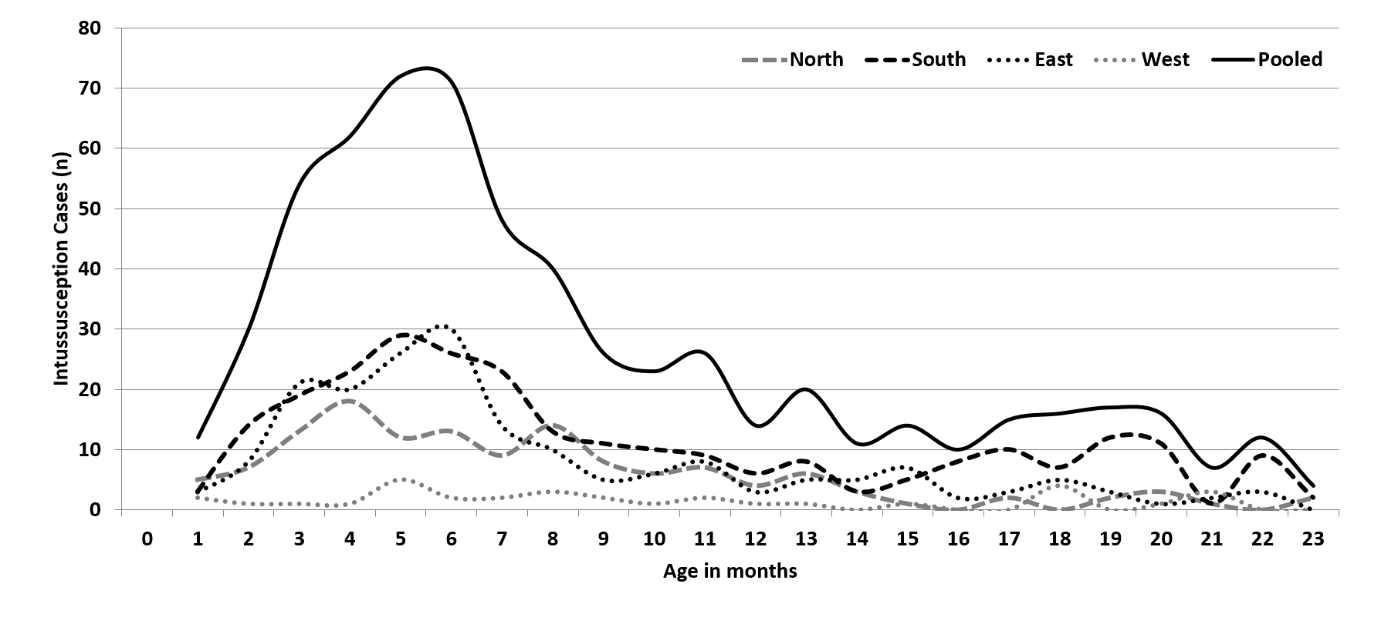


*Note: Age of the children in completed months*

1. Supplementary Table 2: The mode of treatment according to the interval between onset, admission and intervention for the children with intussusception

| Intervals | | Reduction  (n = 306) | Surgery  (n = 245) | Spontaneously resolved ^a^  (n = 69) | No definite treatment ^b^  (n = 1) | P value |
| --- | --- | --- | --- | --- | --- | --- |
| Onset to hospitali-sation | Median  (IQR) | 1  (1, 3) | 2 (1, 3) | 2 (1,4) | 0 (0, 0) | p<0.01** |
|  | ≤1day (%) | 162 (62.3) | 75 (28.9) | 22 (8.5) | 1 (0.4) | p<0.01* |
|  | 2 days (%) | 64  (42.7) | 67 (44.7) | 19 (12.7) | 0 (0) | 0.24* |
|  | 3 days (%) | 40  (41.2) | 48 (49.5) | 9 (9.3) | 0 (0) | 0.17* |
|  | >3days 9%) | 40  (35.1) | 55 (48.3) | 19 (16.7) | 0 (0) | p<0.01* |
|  | Total (%) | 306 (49.3) | 245 (39.5) | 69 (11.1) | 1 (0.2) |  |
| Onset to definite treatment | Median  (IQR) | 2  (1,3) | 3 (2,4) | 2 (1,4) | - | p<0.01** |
|  | ≤1day (%) | 143 (68.8) | 43 (20.7) | 22 (10.6) | - | p<0.01 |
|  | 2 days (%) | 74  (53.6) | 45 (32.6) | 19 (13.8) | - | 0.14 |
|  | 3 days (%) | 42  (38.2) | 59 (53.6) | 9 (8.2) | - | p<0.01 |
|  | >3days (%) | 47  (28.7) | 98 (59.8) | 19 (11.6) | - | p<0.01 |
|  | Total (%) | 306 (49.4) | 245 (39.5) | 69 (11.6) | - |  |
| Hospital stay | Median  (IQR) | 2  (1, 2) | 7 (5, 9) | 3 (2, 5) | 1 (0, 0) | p<0.01** |
|  | 0-5 days (%) | 303 (68.7) | 84 (19.1) | 53 (12) | 1 (0.2) | p<0.01* |
|  | 6-10 days (%) | 2  (1.5) | 124 (91.2) | 10 (7.4) | 0 (0.0) | p<0.01* |
|  | >10 days (%) | 1  (2.3) | 37 (84.1) | 6 (13.6) | 0 (0.0) | p<0.01* |

*Note: IQR: Interquartile range*

** p values estimated by Chi-square test or Fisher’s Exact test, as appropriate*

*** p values estimated by Kruskal-Wallis test*

*^a^ Spontaneously resolved: The patient was managed with IV fluids and nil orally, not required any additional intervention.*

*^b^ No definite treatment: The patient was referred or died before any definite treatment.*

1. Supplementary Table 3: The intussusception cases during risk periods after rotavirus vaccine exposure

| RVV dose (n) | Interval (in days)  Median (IQR) | Number of cases in the periods | | |
| --- | --- | --- | --- | --- |
|  |  | 1-7 days | 8-21 days | 22-60 days |
| RVV 1 (n=96) | 165 (112-258) | 0 | 0 | 5 |
| RVV 2 (n=88) | 134 (78- 200) | 0 | 2 | 13 |
| RVV 3 (n=65) | 99 (38.5-170) | 2 | 8 | 13 |

*Note: Interval: Interval between the immediate last rotavirus vaccine dose receipt and onset of intussusception; RVV: Rotavirus vaccine; IRQ: Interquartile range*

*RVV-1: Rotavirus vaccine dose 1 (Rotavac-68, Rotateq-8, Rotarix- 20)*

*RVV-2: Rotavirus vaccine dose 1 (Rotavac-62, Rotateq-8, Rotarix- 18)*

*RVV-3: Rotavirus vaccine dose 1 (Rotavac-54, Rotateq-3, Rotarix- 8)*

*The children indicated under RVV 1 indicates that the child had received only first dose. Similarly the RVV 2 and RVV 3 indicate the children received two doses and three doses of RVV prior to the intussusception.*

*One child had intussusception on day 0 (day of vaccination itself) and not included in the 1-7 days period.*
